# Congregations and Collections

**Published:** 1889-06-22

**Authors:** 


### The Hospital

A Weekly Institutional Journal of

**Science, Medicine, Nursing, and Philanthropy.**

*For Hospitals, Asylums, Medical Practitioners, Students, Nurses, Families, Parishes, Congregations, and the Charitable Public.*

Vol. VI. No. 143.] [June 22, 1889.

### CONGREGATIONS AND COLLECTIONS.

Many a congregation hangs like a dead weight from a clergyman's neck. He feels that he has done all he can, and said all he can on every topic of public duty, and that he has not succeeded, with all his efforts, in raising his people above the level of a very poor commonplace. If, for once, they should burst the bounds of custom and convention, and do a really noble and generous thing, nobody would be more surprised than their "spiritual pastor." At his best, even the average man has been known to do astonishing deeds of daring or of generosity; but at his ordinary, the unheroic Briton is undoubtedly a commonplace person. It is to be presumed that every man who takes the trouble to go to church has a certain amount of independence and originality of mind; or if not, why is he there? Moreover, the ordinary church-goer must be credited with intelligence enough to understand the value of hospitals, and with sufficient goodness of heart to wish to see them properly supported. In proof of this we have but to cite the history and moderate prosperity of hospitals themselves. Nevertheless, whatever be the present condition of those institutions, the ordinary Englishman never changes his feelings or his contribution to them. Has he been accustomed to give a guinea to the Sunday Fund? Then he will still give a guinea. Are hospitals so rich that they only need fifteen shillings? That is nothing, they may as well have the guinea, he argues. Are they so burdened with extra demands that they do not need one guinea, nor two, but ten from him? It cannot be done: he never has given more than one guinea, and he cannot see his way to doing it now. Surely it might be thought a little variety of some kind would be pleasing to his mind, even if it were in the direction of half-a-guinea, by way of experiment. But that would make too great a demand upon his independent judgment. He has given a guinea for the last twenty years: a guinea he will continue to give: that and no more.

It is hardly fair to the preacher to let him find out, as he soon does, by experience, that his best efforts produce no larger a result than his worst, and his worst hardly any poorer a collection than his best. That is not the way to make him preach either intelligently or earnestly. When a man knows that his labours have but one ending whatever be their merits, he would be more than human if the quality of those labours were not influenced thereby. It is all very well for paterfamilias to make up his mind before he goes to church that he will give a certain amount to the collection. By all means let him resolve that he will give a certain sum, and no less; but let him also do as some people do who have retired from business, but still want to speculate a little. Let him have a few pounds in his pocket, say five or ten, over and above the eminently respectable, but terribly unemotional and uncritical guinea, "to play with," and if the sermon is good, let him give two guineas; if very good, five; but if it has been prepared, and delivered with all the energy of the preacher's brain, and all the fire of his soul, then let the whole ten guineas be poured into the plate, with a regretful sigh that it is not twenty, or even more.

The present, it is said, is a cold and calculating time. Men do not allow their emotions to run away with them. That is not true at all. Men get as excited, as angry, as merry, now, as ever they did, when occasion offers. Was there no excitement at the Derby the other day? Is there never any display of passion in Throgmorton-street? If men would say that they are cool and calculating in their charities because their hearts are not particularly warm in that direction, we could both believe and understand them. But to affirm that the present is a time of general indifference and absence of passionate excitement is the very antipodes of the actual fact. It is a time of profound mental strain and tension, of passion that makes the nerves quiver and the mind give way; only the passion is not aroused by religion, the emotion is not excited by great deeds of benevolence and devoted public service. Nobody thinks it an unnatural thing that Peter the Hermit should have moved every country in Europe and all ranks and classes of the people to hasten with eager feet and panting hearts to the deliverance of the Holy City from the rule of the Infidel Turk. That was a message of war, of carnage, of desolation which the fiery enthusiast proclaimed. Is it not soberly true that the message which religion and science now unite in proclaiming, the message of comfort to the poor, of healing to the sick, of life to the dying, is of tenfold deeper and worthier import than the fantastical and fanatical call of Peter the Hermit, which roused all Europe to frenzy, and cost her tens of thousands of lives? Why then, in the name of instructed, of Christian, and of scientific humanity, should we not now see a mighty wave of noble and passionate sympathy overleaping all bounds of convention and of custom, and pouring into the coffers of charity and benevolence the abundant wealth of a great and generous people? Justice to ourselves, to our worthier instincts, to the memory of our forefathers, who relieved famines, built churches, emancipated slaves, overthrew tyrants, and poured out wealth and life like water when need arose, self-justice calls upon us now to allow our minds to be awakened, our hearts to be quickened into glowing warmth, and our hands to do with all readiness and thoroughness the great deeds which the new time urges upon our consciences. Great deeds are for a great people. They who see great things to be done but will not do them are no longer great, although their ancestors should have been braver than Cæsar and nobler than De Montfort or Washington.

Let the truth be spoken! Forty thousand pounds is a mean sum for the greatest, wealthiest, and proudest city in the world to collect in all its hundreds of temples on a set Sunday. Talk of an annual effort! It is not an "effort" at all. There is no "effort" in it. If ten times forty thousand were collected how many people would be conscious of any injury at the end of the year? The managers of the Hospital Sunday Fund could make the best possible use of four hundred thousand pounds if it were collected. They have never been charged with extravagance or mismanagement. Nobody criticises their operations. Their knowledge, their experience, their economy, their care in distribution have secured for them universal confidence and approbation. What can be said or done to break the ridiculous but injurious spell which limits the Sunday Fund so rigidly to the fixed and immovable sum of forty thousand pounds? It would almost be worth while to burn down the London Hospital in order to make the London public awake from its miserable apathy and give a single hour's original consideration to the merits and the necessities of the noble medical charities of their great metropolis.

